# Protonation of Surface Carboxyls on Rice Straw Cellulose Nanofibrils: Effect on the Aerogel Structure, Modulus, Strength, and Wet Resiliency

**DOI:** 10.1021/acs.biomac.2c01478

**Published:** 2023-04-11

**Authors:** Gabriel D. Patterson, James D. McManus, William J. Orts, You-Lo Hsieh

**Affiliations:** † Bioproducts Research Unit, WRRC, ARS-USDA, Albany, California 94710, United States; ‡ Biological and Agricultural Engineering, University of California, Davis, California 95616, United States

## Abstract

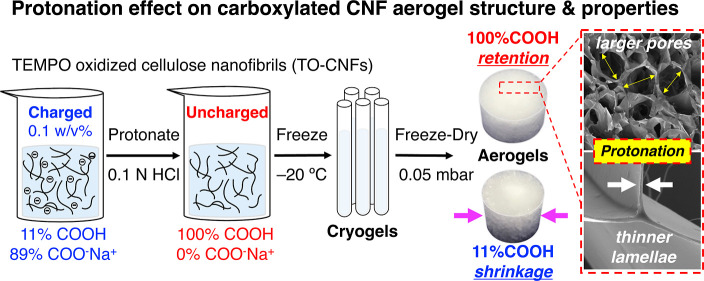

Rice straw cellulose nanofibrils from the optimal 2,2,6,6-tetramethylpiperidine-1-oxyl oxidation/blending process carrying 1.17 mmol/g surface carboxyls were protonated to varying charged (COO^–^Na^+^) and uncharged (COOH) surfaces. Reducing the electrostatic repulsion of surface charges by protonation with hydrochloric acid from 11 to 45 and 100% surface carboxylic acid most prominently reduced the aerogel densities from 8.0 to 6.6 and 5.2 mg/cm^3^ while increasing the mostly open cell pore volumes from 125 to 152 and 196 mL/g. Irrespective of charge levels, all aerogels were amphiphilic, super-absorptive, stable at pH 2 for up to 30 days, and resilient for up to 10 repetitive squeezing-absorption cycles. While these aerogels exhibited density-dependent dry [11.3 to 1.5 kPa/(mg/cm^3^)] and reduced wet [3.3 to 1.4 kPa/(mg/cm^3^)] moduli, the absorption of organic liquids stiffened the saturated aerogels. These data support protonation as a critical yet simple approach toward precise control of aerogels’ dry and wet properties.

## Introduction

Cellulose nanofibrils (CNFs) have been commonly produced by shear force processing of aqueous suspensions of cellulose that is often chemically pretreated to aid fibrillation into homogeneous dispersions. The presence of charges plays an essential role in facilitating the disintegration of cellulose into nanocelluloses and the dispersion of the resulting nanocelluloses in aqueous media. The presence of charges in the fabrication and dispersion of nanocelluloses is well recognized and has been extensively studied in CNFs by 2,2,6,6-tetramethyl piperidine-1-oxyl (TEMPO)-mediated oxidation.^1^ To process or convert TEMPO-oxidized CNFs (TO-CNFs) into solids, a significant quantity of water must be removed where the concentration,^2−4^ degrees of oxidation,^3,5^ surface charge,^3,5^ and protonation^5−7^ have been shown to influence their structures and, in particular, their wet mechanical properties and performances.^5,8,9^

TEMPO-mediated oxidation of purified rice straw (RS) cellulose, followed by blending, has produced TO-CNFs in reduced dimensions and increased surface charges with increasing oxidation levels.^10^ From optimal TEMPO oxidation (5 mmol NaClO/g cellulose) and blending (37k rpm, 30 min), RS cellulose was readily fibrillated into ca. 2 nm wide and 1 μm long TO-CNFs with 1.29 mmol/g charges at 97% yield. Rapid ice nucleation (−196 °C, 10 m) and lyophilization of TO-CNF suspensions at and below 0.1 w/v % concentrations produced viable self-assembled fibrils irrespective of their carboxylation levels. However, the finest and most uniform sub-micrometer fibrils were more favorably assembled from unoxidized CNFs by blending^10^ and aqueous counter collision,^11^ less oxidized with low surface carboxyls^10^ and highly dissociated carboxylates,^2^ up to relatively low concentrations.

In contrast, slowly freezing (−20 °C, 15 h) TO-CNFs at higher concentrations (0.2–0.6 w/v %) nucleated much larger ice crystals to further concentrate TO-CNFs toward extensive association and, upon lyophilization, produced an interconnecting-thin-film-like cellular wall structure surrounding large pores vacated from the sublimation of ice crystals in the form of ultra-low-density aerogels.^2^ While surface charges have affected TO-CNF organization in films due to varying oxidation levels,^5^ or different levels of protonation under the same oxidation,^7^ how surface charges, or the lack thereof, affect the self-assembly of TO-CNFs in aerogels via slow ice-templating and lyophilization has not been elucidated.

Fully hydrated (wet) nanocellulose-based materials are prone to macroscopic disintegration, reportedly due to water uptake into spaces between self-assembled solids and enhanced by the osmotic swelling pressure from charged surface groups and their counterions.^9^ While the free volume in semicrystalline polymers is associated with amorphous regions,^12^ the spaces in self-assembled nanocellulose structures are attributable to the interfacial spaces, such as 0.47 nm-diameter pores in films from 0.15 w/v % TO-CNF by positron annihilation lifetime spectroscopy^13^ or 10 nm intra-lamellar mesopores in aerogels from 0.6 w/v % TO-CNF by N_2_ desorption.^14^ It is plausible that the water uptake into the inter-nanofibril spaces within the self-assembled cellular walls exacerbated the wet mechanical properties and performances of aerogels reported previously.^3,15,16^ While much effort has been made to improve the wet mechanical properties of charged CNF aerogels, such as by chemical cross-linking,^15,16,18,19,22,23^ electrostatic interaction,^17^ coupled solvent exchange, and super-critical CO_2_-drying,^20,21^ how the surface charges of carboxylated CNFs affect their self-assembly and the interfacial property in aerogels has not been established. Specifically, understanding how surface charges of carboxylated CNFs affect their interfacial association within aerogel structures is fundamentally critical and may serve as a frontline approach to improve aerogel structures, interactions with liquids, and their behavior in water.

The central hypothesis herein was that the ability of TO-CNFs to self-organize and self-assemble into aerogels is dictated by their proximity and charged surfaces and interfaces during ice-templating and lyophilization, which thereby control their morphological structures and mechanical properties. Reducing electrostatic inter-TO-CNF repulsion by protonation may minimize interfacial spaces, thereby enhancing close and compact associations of TO-CNFs toward a more tenacious aerogel structure with improved wet properties.

TO-CNFs from one TEMPO oxidation and mechanical blending condition with an optimal level of carboxylation and consistent dimensions were protonated to convert the carboxylate (COO^–^Na^+^) into varying amounts of carboxylic acid (COOH), followed by slow freezing (−20 °C) and lyophilization to produce aerogels. The self-assembled aerogels from TO-CNFs with varying surface carboxylic acid (% COOH) to carboxylate (COO^–^Na^+^) contents were characterized in terms of their pore morphology, crystallinity, thermal stability, surface chemistry, liquid absorption, and dry and wet compressive toughness and strength to delineate the effect of protonation on these structure–property–function relationships.

## Experimental Section

### Materials

Cellulose was purified from Calrose variety RS by sequential organic toluene/ethanol extraction, 1.4% NaClO_2_ (pH 3.5, CH_3_COOH), and 5% KOH to ca. 36% yield.^24^ Hydrochloric acid (HCl, 1 N, Certified, Fisher Scientific), sodium hydroxide (NaOH, 1 N, Certified, Fisher Scientific), sodium hypochlorite solution (NaClO, 11.9%, reagent grade, Sigma-Aldrich), 2,2,6,6-tetramethylpiperidine-1-oxyl (TEMPO, 99.9%, Sigma-Aldrich), sodium bromide (NaBr, BioXtra, 99.6%, Sigma-Aldrich), decane (C_10_H_22_, Certified ACS, Fisher Scientific), and chloroform (CHCl_3_, HPLC grade, EMD) were used as received. All water (pH 5.7) was from the Milli-Q water purification system (Millipore Corporate, Billerica, MA).

### TEMPO-CNFs, Protonation of Surface Carboxyls, and Assembly into Aerogels

Optimized TEMPO-mediated oxidation (5 mmol/g NaClO, pH 10) followed by shearing by high-speed blending (37.5k rpm, 30 min, Vitamix 5200, 250 mL) of RS cellulose (1.0 g) produced cellulose nanofibrils (TO-CNFs) at 0.4 w/v % in the aqueous supernatant via centrifugation (5k rpm, 15 min, ThermoFisher Megafuge 1.6 L) as per our previous report.^10^ The aq. TO-CNFs (pH of 5.7) were divided into three aliquots and diluted to 0.13 w/v % below the 0.2 w/v % gelation concentration. One was the as-is aq. TO-CNFs, and the other two were protonated by dropwise additions of dilute HCl (0.1 M) to pH 4 and 2.8, respectively, and maintained under vigorous stirring for 1 h to prevent flocculation, then dialyzed to below 10 μ*S*/cm. The three parallel aliquots of aq. TO-CNFs had different percentages of sodium carboxylate and carboxylic acid contents at the same degree of carboxylation. Each aq. TO-CNF suspension was degassed, concentrated (Buchi Rotavapor R-114) to 0.6 w/v %, pipetted (∼9 mL) into 1.4 cm inner diameter (ID) Pyrex borosilicate glass or polypropylene (PP) tubes to ca. a height of about 6 cm, and then frozen (−20 °C, 15 h, 6 mL) and freeze-dried (−50 °C, 0.05 mbar, 3 days, Free Zone 1.0 L Benchtop Freeze Dry System, Labconco, Kansas City, MO) to produce aerogels.

### Characterizations

The degree of carboxylation on CNFs by TEMPO oxidation was determined by acid–base conductometric titration (OAKTON pH/Con 510 series). The original aq. CNF aliquot obtained at pH 5.7 was separated into 50 mL aliquots at a concentration of 0.05 w/v %, and 200 μL of 0.5 M NaCl was added to raise the conductivity and 50 μL of 1 N HCl to convert all surface carboxyls to carboxylic acids with excess H^+^ in the dispersion. This acid–base titration used 0.01 M NaOH as the titrant, added in 100 μL increments. The titration curve plotting conductivity against added NaOH showed three distinct regimes, i.e., an initial negative slope of consumed free acid, a plateau region representing neutralized surface carboxylic acid to sodium carboxylate, and a positive slope indicating excess NaOH. The total carboxyl content (σ, mmol/g cellulose) was calculated as
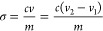
1where *c* is the NaOH concentration (M), *m* is the CNF mass in the suspension (g), and *v* is the volume (mL) of NaOH consumed in the plateau region from *v*_1_ to *v*_*2*_*.* Since all three CNF aliquots had the same degree of carboxylation, the percent of carboxylic acid on each of the three CNF aliquots was determined similarly without adding HCl. Thus, titration with NaOH converted only surface carboxylic acids on CNFs, and the titration curve showed just the second and third regimes.

The nanoscale dimensions of CNFs were determined using atomic force microscopy (AFM) (Asylum-Research MFP-3D, OMCL-AC160TS standard silicon probes, 1 Hz) for the thickness (*n* > 100) and transmission electron microscopy (TEM) (JEOL 1230, 100 kV) for the width (*n* > 25). Both utilized just 10 μL of 0.0005 w/v % as-is CNF (pH 5.7) air-dried on fresh mica for AFM and glow-discharged carbon-coated grids (300-mesh copper, formvar-carbon, 100 kV, Ted Pella Inc., Redding, CA) stained with 2 w/v % uranyl acetate for TEM. The TO-CNF lengths were estimated from AFM images of individualized nanofibrils.

The diameter (cm), weight (mg), volume (cm^3^), and density (ρ_a_, mg/cm^3^) of 1 cm tall cylindrically shaped aerogel sections were determined, and their percent porosity (Φ, %) was calculated as
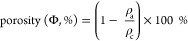
2where ρ_c_ is the density of cellulose (1.6 g/cm^3^).^25^ The total pore volume (*V*_p_), indicative of the absorption capacity of the aerogel, was calculated using the density (ρ_a_, mg/cm^3^) and porosity (Φ, %) by
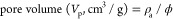
3while the measured liquid absorption (mL/g) was simply the volume of liquid retained in the aerogel after full immersion, or
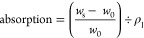
4where *w*_s_ and *w*_0_ were the weights of saturated and dry aerogel, and ρ_l_ was the liquid density (g/mL). Welch’s *T*-tests (*a* = 0.05) of unequal variance were applied for the statistical comparison of the means. The percent (%) of measured absorption over the pore volume or absorption capacity was also reported.

Fourier transform infrared (FTIR) spectroscopy was conducted on milled aerogel powders pressed into pellets (1:100, w/w KBr) (Thermo Nicolet 6700, RT, 4 cm^–1^ resolution). Freeze-dried cellulose (−196 °C, 0.1 w/v %) and milled aerogel powders were characterized by thermogravimetric analysis (TGA-50, Shimadzu, Japan, 10 °C/min, 50 mL N_2_/min) and the degree of (X-ray diffraction) crystallinity (PANalytical XRD, Ni-filtered Cu Kα, λ = 0.1548 nm, 45 kV, 40 mA). The crystallinity index (CrI) was determined using the Segal et al. equation on specific peaks in the X-ray diffractograms, whereby
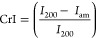
5where *Ι*_200_ was the peak intensity of the 200 lattice plane located at 22.7° 2θ, and *Ι*_am_ is the intensity minimum at ca. 18.7° 2θ, attributable to non-crystalline or amorphous cellulose *Ι*.^26^

The micromorphology of each CNF aerogel was characterized using a field-emission scanning electron microscope (Thermo Fisher QUATTRO S Environmental SEM-FEG, 10 mm WD, 5 kV). The aerogels were cut into radial and longitudinal cross-sections using a new razor blade. The cross-sections were then sputter-coated (Au, 20 mA, 60 s, <10 nm thickness) (BioRad SEM Coating System) and imaged by SEM. The cell wall thickness (nm, *n* > 30) and pore widths (μm, *n* > 30) were measured using the SEM software. Compressive stress–strain tests characterized the dry and wet mechanical properties of each aerogel using 1 cm tall sections placed between two glass plates for measurements in the air (21 °C, 65% relative humidity) and between a glass plate and a jar containing water (pH 5.7) (Instron 5566, 2.5 kN load cell, 1 mm/min loading–unloading). The Young’s modulus of each aerogel in the air and the water was calculated from the stress–strain linear elastic region, and the scaling relationship with density was calculated by
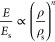
6where *E* and ρ were the respective modulus and density of each aerogel, and *E*_s_ (∼150 GPa) and ρ_s_ (1.6 g/cm^3^) were the longitudinal modulus and bulk density of cellulose.^27^

## Results and Discussion

### CNFs and Protonation

**Figure 1:**
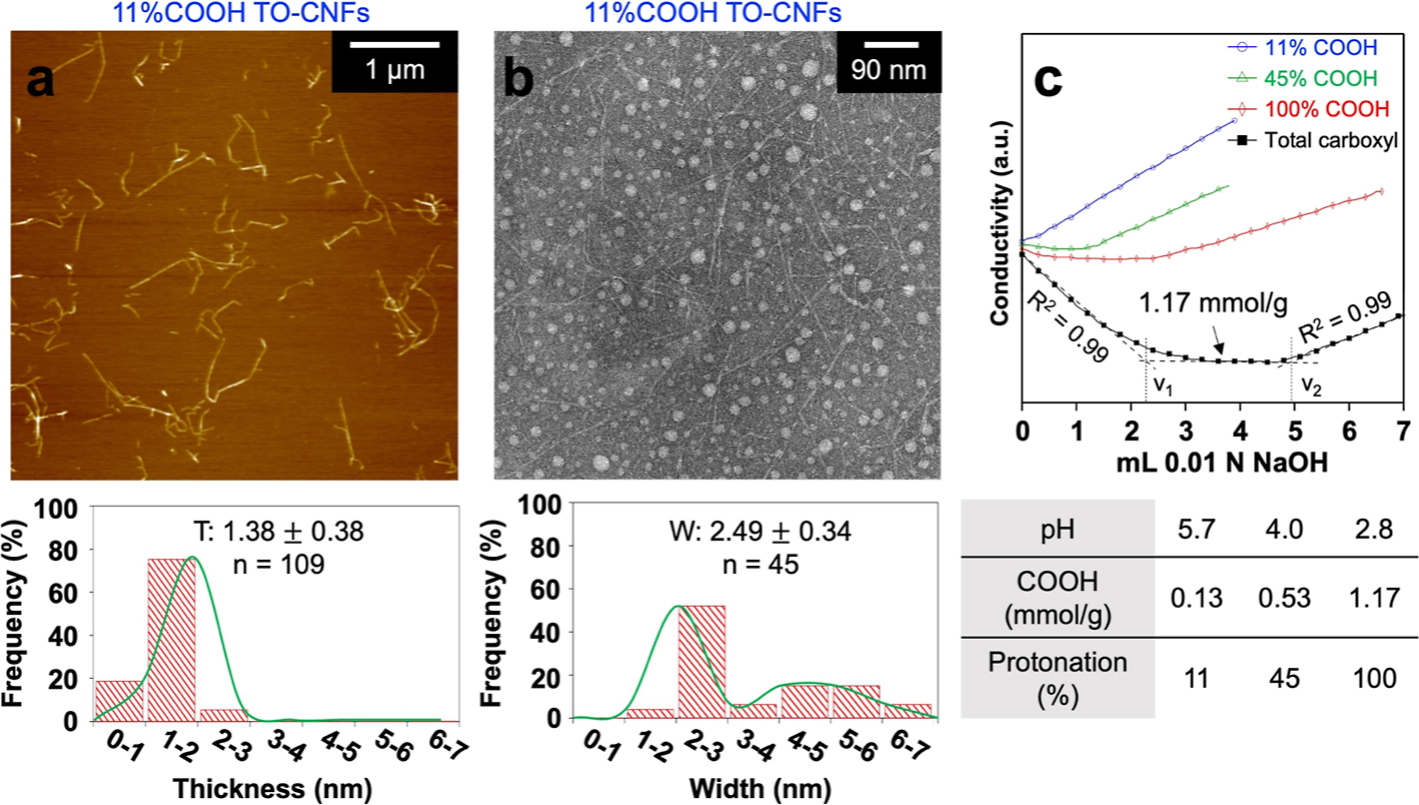
Characteristics of TO-CNFs: (a) thickness (*T*, top) and distribution (bottom) by AFM (0.0005 w/v %); (b) width (*W*, top) and distribution (bottom) by TEM (0.0005 w/v %); (c) acid–base titration (0.05 w/v %) of three varied protonated aliquots (top) and a summary of COOH and protonation of surface COOH/COO^–^Na^+^ (bottom).

Regioselective TEMPO-mediated oxidation at an optimal 0.81:1 NaClO/AGU molar ratio followed by 30 min of blending^10^ converted 95% of RS cellulose into 0.4 w/v % C6 TO-CNFs with a 1.38 ± 0.38 nm thickness, a 2.49 ± 0.34 nm width, and ca. a 1 μm length (Figure 1a,b). The aqueous TO-CNF dispersion was divided into three equal volume portions; one “as-is” (pH 5.7) while the other two were protonated with 0.1 M HCl to either 4 or 2.8 pH and then dialyzed in water. Acid–base conductometric titration of the as-is TO-CNF dispersion showed a total surface carboxylation of 1.17 mmol/g, of which 0.13 mmol/g was carboxylic acid (COOH), thus was designated as 11% COOH TO-CNF. The two protonated samples at respective pHs 4 and 2.8 showed 0.53 and 1.17 mmol/g COOH to be designated as 45% COOH and 100% COOH TO-CNFs. These three aqueous aliquots of 11%COOH, 45%COOH, and 100% COOH TO-CNFs had the same degree of carboxylation and morphology but 89, 55, and 0% negative sodium carboxylate (COO^–^Na^+^) surface charges, respectively. All three aliquots showed shear-thinning behaviors, yet the opacity increased with increasing protonation, which was consistent with the reduced optical transmittance and slight aggregation of fully protonated TO-CNFs shown previously.^7^ In the absence of the inter-TO-CNF electrostatic repulsion, the uncharged 100% COOH TO-CNF was most viscous, attributable to significant hydrogen bonding association among C6 carboxylic acids and abundant C2 and C3 hydroxyls, in addition to physical entanglements due to the high L/W aspect ratio of TO-CNFs. The rheological changes were similar to the most-viscous and least sulfonate surface charge relationship of rod-like cellulose nanocrystals,^28^ corroborating more significant inter-nanocellulose associations and closer proximity with lower or no surface charges.

### Aerogel Properties

**Figure 2:**
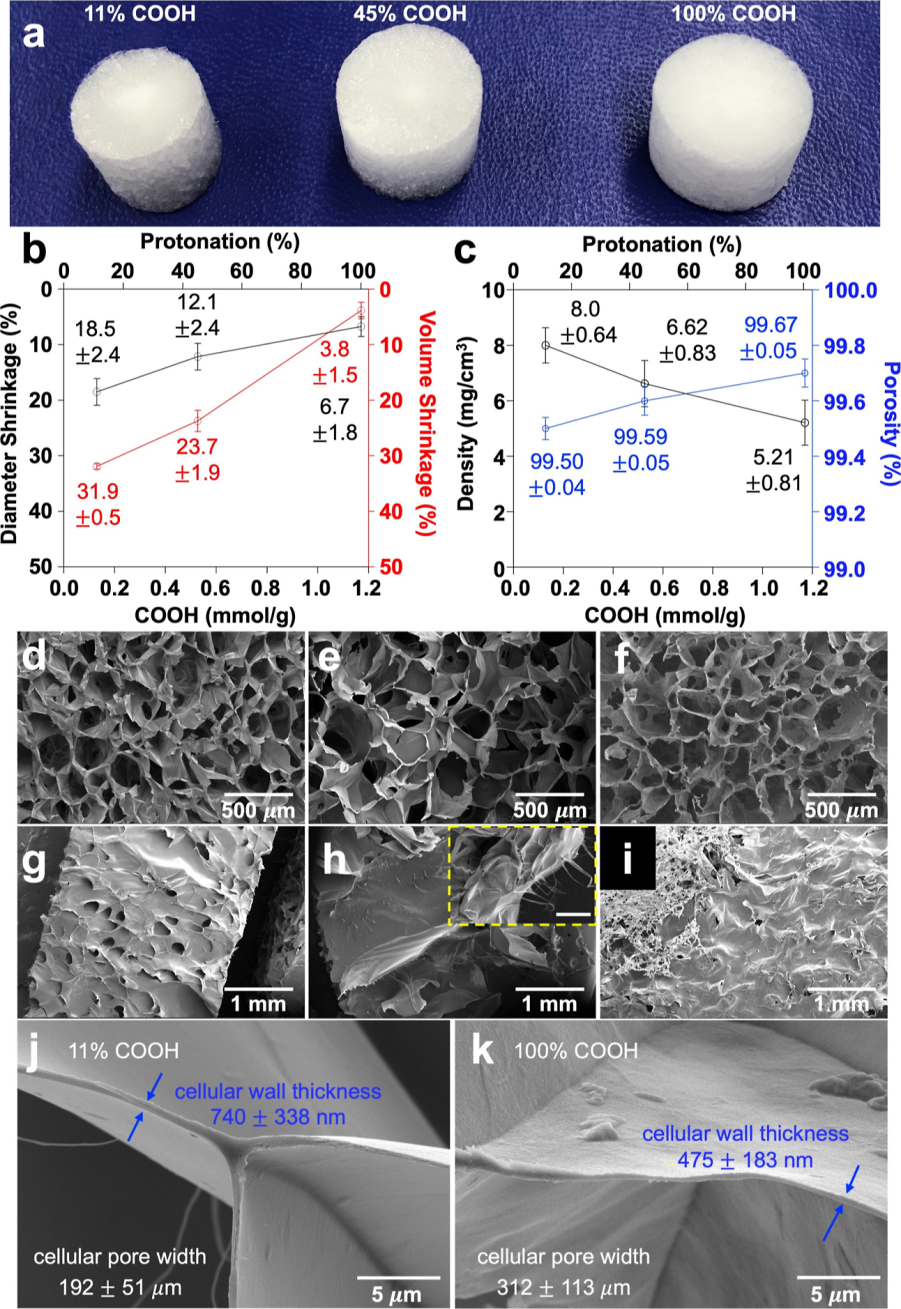
Characteristics of aerogels from TO-CNFs at three levels of protonation: (a) photographic images; (b) diameter and volume; (c) density and porosity; SEM images of radial cross-sections of aerogels from (d) 11%COOH, (e) 45%COOH, and (f) the 100% COOH protonated TO-CNFs; (h-i) corresponding external surfaces; (j,k) lamellae (pore wall) thickness and pore size; and (h, insert) radial view of the 45% COOH TO-CNF external surface (scale bar: 100 μm).

At a fixed 1.17 mmol/g total surface carboxylation and 0.6 w/v % TO-CNF concentration, the aerogels fabricated in the 1.4 cm ID glass tubes maintained their original height but had reduced diameters of 1.14, 1.23, and 1.31 cm or 18.5, 12.1, and 6.7% shrinkage, from 11%COOH, 45%COOH, and 100% COOH TO-CNF aliquots, showing 31.9, 23.7, and 3.8% volumetric shrinkage or 68.1, 76.3, and 96.2% volume retention (Figure 2a,b). The pronounced shrinkage from suspension-to-aerogel was attributed to the repulsion of anionic surface-charged TO-CNFs away from the negatively charged glass surface, creating a boundary layer of water near the glass with little or no TO-CNF. The fully protonated TO-CNF aerogel, i.e., 100% COOH TO-CNF, showed the best shape and volume retention, supporting minimal charge-induced TO-CNF excluded volume. Hence, the aerogel from the most-charged 11% COOH TO-CNFs with the most volumetric shrinkage was the densest (8.0 mg/cm^3^) and least porous (99.5%) (Figure 2c). In a highly linear relationship (*r* = 0.991, *p* = 0.086), reducing surface charges by protonation to 45% COOH and 100% COOH TO-CNFs gave aerogels with reduced densities of 6.6 and 5.2 mg/cm^3^ and increased porosities of 99.6 and 99.7%, respectively. While volumetric shrinkage of aerogel was expected of charged TO-CNFs, the 15% reduction of the aerogel by the most charged 1.04 mmol/g TO-CNF here was significantly less than the 25% shrinkage of aerogel from far less charged 0.5 mmol/g carboxymethyl CNFs reported.^29^

Irrespective of the levels of protonation or surface charges on TO-CNFs, all three aerogels had similar morphologies, i.e., thin and smooth film-like cellular walls surrounding ca. 200 to 300 μm wide and hexagonally shaped pores (Figure 2d–f). Conversely, the external surfaces of these aerogels did vary, i.e., the mostly charged 11% COOH TO-CNF aerogel had large surface pores that appeared to be interconnected with the internal pore structure and attributable to significant ice crystal growth by water in the TO-CNF-excluded boundary. The partially charged 45% COOH TO-CNF aerogel showed the most heterogeneous surface morphology with pores, smooth sections, and ridges. The uncharged 100% COOH TO-CNF aerogel had a smooth and non-porous surface as if mirroring the glass surface, corroborating its minimal TO-CNF-excluded volume (Figure 2g–i). Closer examination revealed that the mostly charged 11% COOH TO-CNF aerogel had the thickest (740 ± 338 nm) cellular walls and the smallest (192 ± 51 μm) pores, while the uncharged 100% COOH TO-CNF aerogel had 36% thinner walls (475 ± 183 nm) and 60% larger pores (312 ± 113 μm) (Figure 2j,k). As salts are known to impact ice crystal growth by disrupting hydrogen-bonding networks,^31^ larger ice crystals are expected in the uncharged 100% COOH TO-CNF aerogel, leaving slightly larger pores and porosity and more compacted cellular walls. The thicker cellular walls of the 11% COOH TO-CNF aerogel support that electrostatic inter-TO-CNF repulsion, which impedes close inter-fibrillar associations. Upon freezing and lyophilization, those intra-cell wall spaces or free volume spaces are consistent with our previously reported intra-lamellar 10 nm mesopores.^14^ Since all three TO-CNFs had the same level of carboxylation, nanoscale dimensions, and fixed 0.6 w/v %, only eliminating the surface charge by protonation was responsible for tighter inter-nanofibril compaction of the cellular walls in the 100% COOH TO-CNF aerogel.

**Figure 3:**
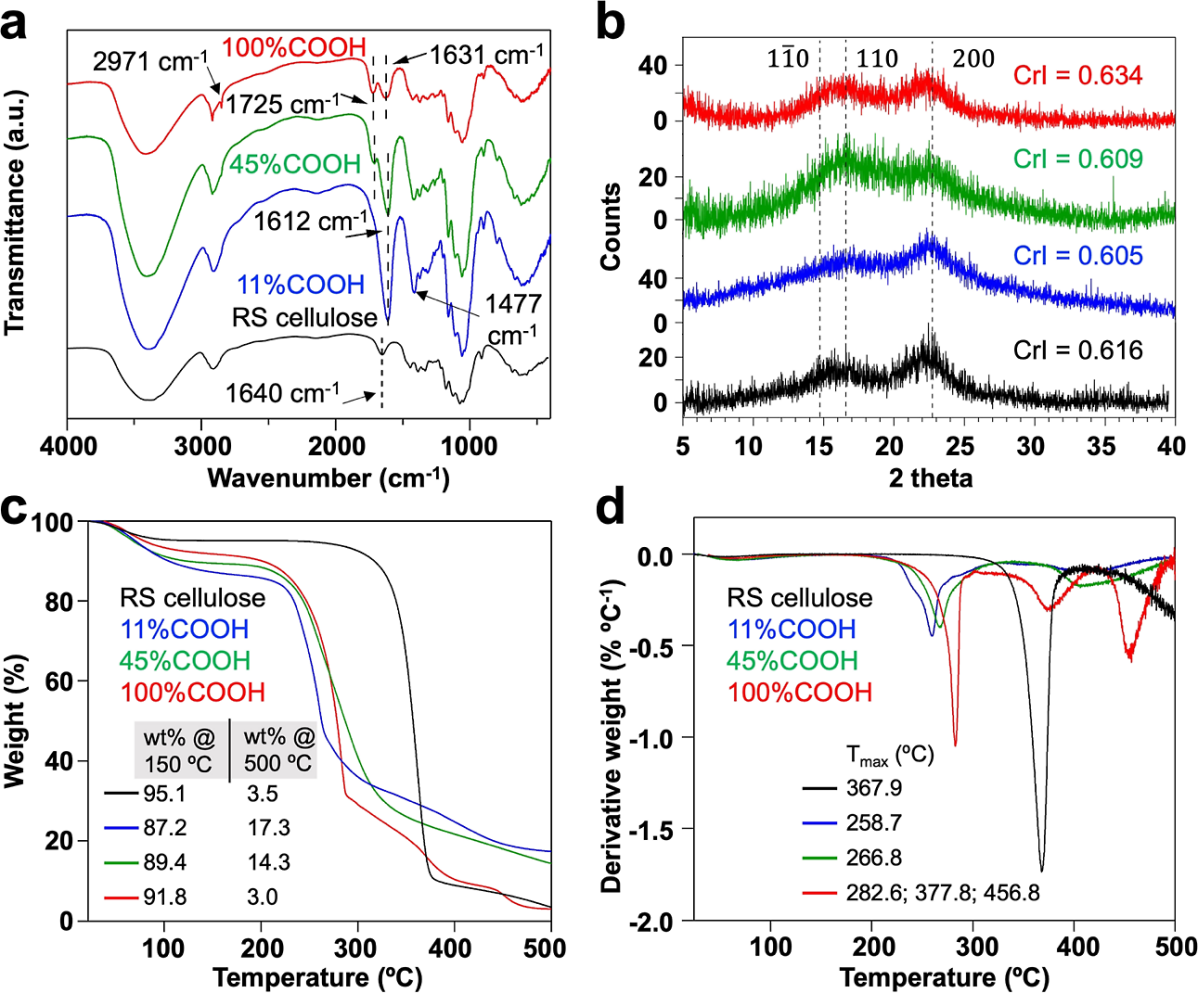
Characterization of TO-CNF aerogels (−20 °C, 0.6 w/v %): (a) FTIR, (b) XRD, (c) TGA, and (d) dTGA of cellulose (black), and TO-CNF at 11% (blue), 45% (green), and 100% COOH (red).

FTIR spectroscopy showed the 1612 cm^–1^ C=O stretching band (overlapping with the absorption of O–H deformations) and the 1477 cm^–1^ C–O stretching band of dissociated sodium carboxylate groups of the mostly charged 11% COOH aerogel, confirming the effect of TEMPO oxidation. The 1725 cm^–1^ C=O stretching and 2971 cm^–1^ O–H stretching peaks of the 100% COOH aerogel accompanied the re-emergence of the 1631 cm^–1^ O–H deformation of water vapor, which confirmed the effect of protonation (Figure 3a). Notably, the distinct C=O stretching band at 1725 cm^–1^ signified hydrogen-bonding carboxylic acid groups,^6,30^ while isolated and non-hydrogen-bonding carboxylic acid was found at ca. 1740 cm^–1^ as with the open-ring and carboxylated periodate-chlorite-oxidized CNFs.^3^ Hence, the uncharged 100% COOH TO-CNFs could self-organize randomly via hydrophilic and hydrophobic interactions without surface charges upon freezing/freeze-drying.

The 100% COOH TO-CNF aerogel had the highest CrI of 0.634 compared to the slightly lower 0.605 and 0.609 CrI of the respective 11% COOH and 45% COOH TO-CNF aerogels and the 0.616 CrI of the original cellulose (Figure 3b). The reduced CrI values for the aerogels from the as-is and partially protonated TO-CNF were expected from TEMPO oxidation and shear force blending. At the same time, the increased CrI of the 100% COOH TO-CNFs gave evidence of interfacial crystallization. The uncharged surface carboxylic acid is expected to enhance the proximity of CNFs with little inter-CNF free volume to self-assemble compactly, to form hydrogen bonds, and to recrystallize. The 11% COOH TO-CNF aerogel had the highest moisture content and the lowest *T*_max_ attributable to porous, cellular walls—consistent with enhanced water uptake by the charged groups and their counterions^9^—and a higher specific surface to heat exposure, respectively. In contrast, the lowest moisture and higher *T*_max_ of the 100% COOH TO-CNF aerogel supported the notion of tenaciously assembled lamellae via compact, extensively hydrogen-bonded, and possibly recrystallized TO-CNF interfaces (Figure 3c,d).

**Figure 4:**
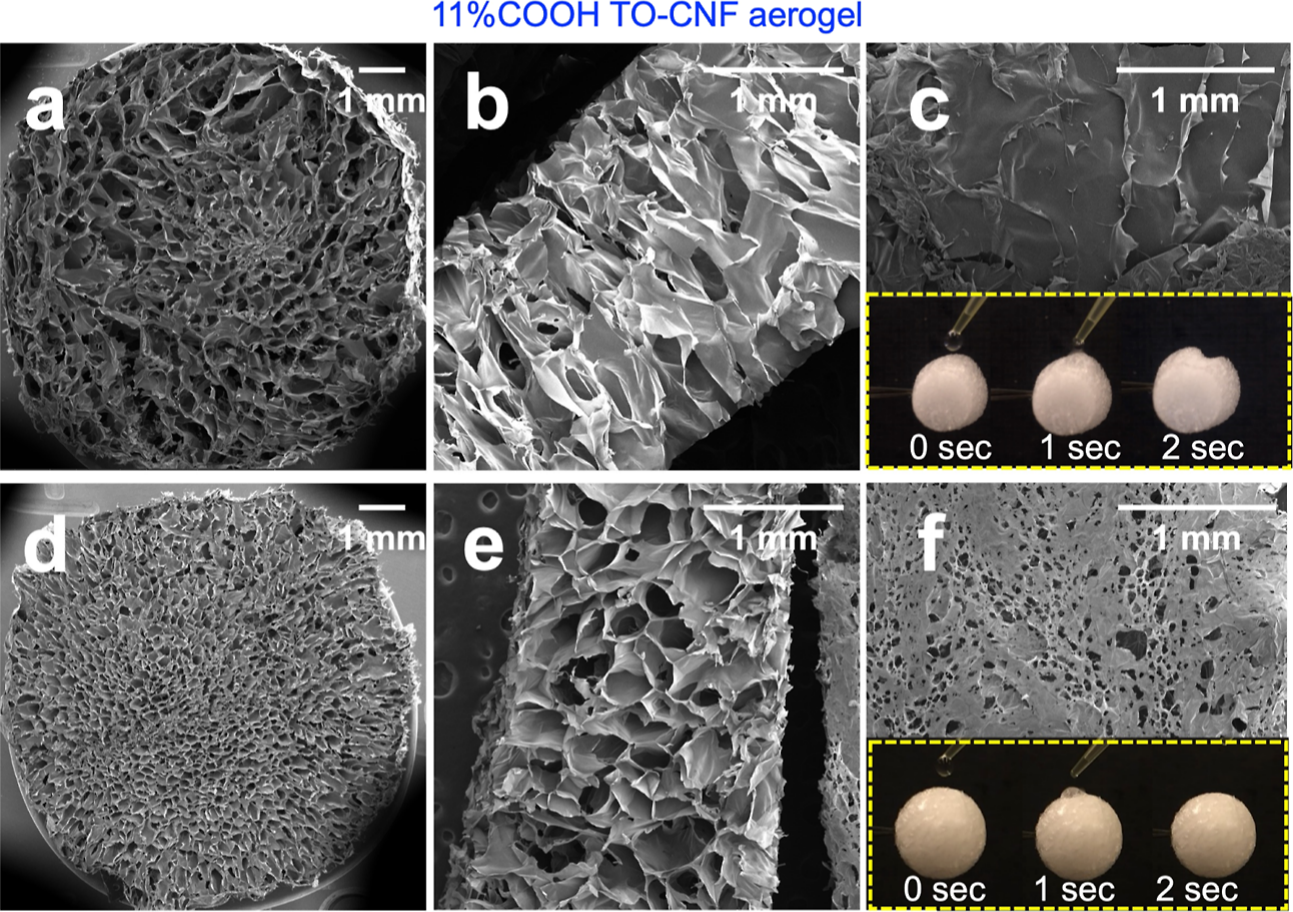
SEM of the as-is 11% COOH TO-CNF aerogel formed in PP (a–c) and borosilicate glass (d–f) tubes: (a,d) radial cross-sections, (b,e) longitudinal cross-sections, and (c,f) external surfaces and wetted with a 10 μL water droplets.

As TO-CNF charges affected the aerogel densities and porosities due to charge repulsion against the glass, the uncharged and hydrophobic PP tubes were investigated for aerogel from the most highly charged 11% COOH TO-CNFs. The PP-molded aerogels had a statistically equal density of 8.1 mg/cm^3^ (±0.7) as the glass-molded aerogel (8.0 ± 0.6 mg/cm^3^) (*p* > 0.05, Welch’s *T*-Test). However, the PP-molded aerogel had anisotropic and heterogeneous pore sizes and shapes in both radial and longitudinal directions (Figure 4a,b); i.e., the outer pores were most irregular and largest (ca. 1 mm width) and became incrementally smaller and more compact toward the center. Furthermore, the external surface of the PP-molded aerogel was non-porous and had distinctive spin-like features (Figure 4c), in stark contrast to the isotropic, honeycomb-like, and an interconnecting open cell structure with a porous external surface of the glass-molded aerogel (Figure 4d–f). The concentrically reducing pore sizes of the aerogel were attributed to the more thermally insulating and hydrophobic PP tube while also explaining the inward collapse of the aerogel surface when wetted with a water droplet driven by increasing capillary pressure (Figure 4c,f). It is worth noting that the water absorption of fully immersed and saturated aerogels from the PP- and glass-molded 11% COOH TO-CNF aerogels was statistically equal (*p* > 0.05, Welch’s *T*-Test) at approximately 104 mL/g water, filling 80% of the total ∼130 cm^3^/g pore volume. Hence, the mold materials affect the ice templating and, thus, the pore morphology and surface wetting behavior of a negatively charged CNF aerogel, but not their overall porosity and absorption capacity.

### Amphiphilic Super-absorption

All three aerogels were amphiphilic and super-absorptive, rapidly taking in water, decane, and chloroform in <3 s. The absorption of water (103–166 mL/g), decane (78–163 mL/g), and chloroform (86–154 mL/g) increased linearly with increasing pore volumes of 125, 152, and 196 mL/g for the 11% COOH, 45% COOH, and 100% COOH TO-CNF aerogels, respectively (Table 1). These liquid absorptions filled 62 to 85% of the pore volumes, confirming the largely open cell and interconnected pore structure observed by SEM (Figures 2, 4). The most charged 11% COOH TO-CNF aerogel absorbed more water than decane or chloroform, indicating a more significant proportion of hydrophilic cellular wall surfaces. In contrast, similar polar and nonpolar liquid absorption by the 45% COOH and 100% COOH TO-CNF aerogels signified similar proportions of hydrophilic and hydrophobic surfaces. Such was ascribed to the random self-organization of TO-CNFs with low or no charges such that their inter-facial self-assembly was stabilized by polar and nonpolar associations or hydrogen bonding and van der Waals interactions, respectively.

**Table 1:** Liquid Absorption of Aerogels from CNF with Varied Levels of Protonation

aerogel	density	porosity	absorption capacity	liquid absorption^a^					
				water		decane		chloroform	
	(mg/cm^3^)	(%)	(mL/g)	(mL/g)	(%)	(mL/g)	(%)	(mL/g)	(%)
11%COOH	8.0	99.5	125	103	82	78	62	86	69
45%COOH	6.6	99.6	152	117	77	119	78	113	74
100%COOH	5.2	99.7	196	166	85	163	83	154	79
*r*^b^			0.999	0.969		0.999		0.994	

a Liquid absorption is expressed as both mL/g and % of the total pore volume or absorption capacity.

b The linear regression of liquid absorption and percent carboxylic acid content.

**Figure 5:**
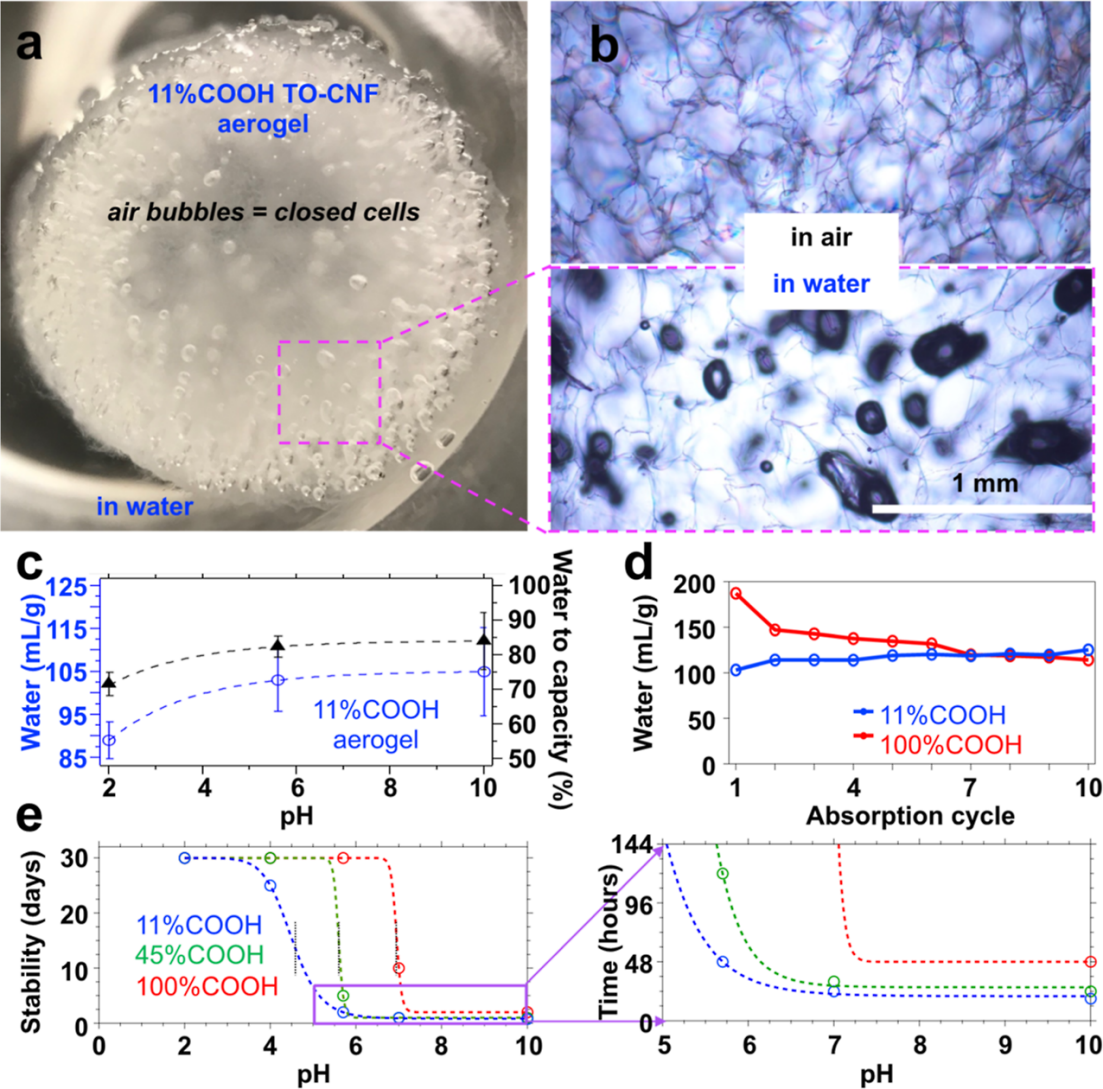
11% COOH TO-CNF aerogel: (a) image of a fully water-immersed aerogel; (b) optical microscopy of the radial cross-section in the air (top) and the water (bottom); (c) pH-dependent water absorption. Water absorption: (d) cyclic (pH 5.7); (e) pH-dependent wet stability in days and hours (expanded).

A fully water-saturated aerogel from the most charged or least protonated 11% COOH TO-CNF was also titrated to show no charge, indicating the charged carboxylates and their counterions to be embedded within the cellular walls and corroborating the inter-TO-CNF free volume or porosity within the cellular walls, which is consistent with the 10 nm mesopores by N_2_ desorption of the highly charged (86%) and carboxylated (1.29 mmol/g) TO-CNF aerogel.^10,14^ Ostensibly, protonation would reduce inter-nanofibril spacing toward higher nanofibril compaction in the cellular wall. Furthermore, the incomplete filling of the entire pore volume of the aerogels indicated some degree of closed cell pore structure regardless of the protonation extent and the liquids. Further evidence of closed cells was visualized as trapped air bubbles within a fully immersed and saturated aerogel (Figure 5a,b).

The most charged 11% COOH TO-CNF aerogel absorbed less water at pH 2, i.e., 89.5 mL/g or 72% of capacity, than at pH 5.7 and 10, or 103 and 105 mL/g (ca. 85% of capacity), respectively (Figure 5c), indicating constriction of inter-nanofibrillar spaces in the cellular walls by protonation at pH 2. Hence, fewer closed cells are accessible and interconnected with the primary open cell pore morphology through nanopore channels within cell walls at pH 5.7 and 10. The pH-dependent water absorption strongly supports the role of the embedded charged groups and their counterions in creating cellular wall porosity, which was previously associated with water uptake into charged and non-crystalline domains^8,9^ and, now, nanoscopic pore interconnectivity and transport.

Repetitive water absorption by squeezing or compressing (ε > 90%) up to 10 cycles trended oppositely for the as-is and fully protonated TO-CNF aerogels, i.e., from an initial absorption of 103 and 187 mL/g, respectively, to converge at 119 mL/g at the seventh absorption, then slightly higher absorbed by the as-is aerogel (125 mL/g) than the fully protonated aerogel (114 mL/g) at the 10th absorption (Figure 5d). The denser (8.0 mg/cm^3^) as-is aerogel exhibited better water-activated shape recovery following squeezing and absorbed 21% more by the 10th cycle, indicating the opening of some closed cells and inter-nanofibril spaces within the cellular walls. Conversely, the nearly 40% reduction in water absorption of the fully protonated aerogel from the 1st to the 10th cycle was consistent with its lower density (5.2 mg/cm^3^) and less wet-resilient structure.

Regardless of the protonation-dependent shape recovery property, all aerogels showed excellent wet stability under acidic condition of pH 2, remaining intact for at least 30 days (Figure 5e). Protonation of surface-charged TO-CNFs prior to self-assembly into aerogels further improved pH stability in near-neutral to even basic conditions, i.e., the fully protonated TO-CNF aerogel remained intact at pH 5.7 and 10 for 30 and 2 days, respectively, while the as-is TO-CNF aerogel disintegrated within 48 h at pH 5.7 and 12 h at pH 10. Hence, while surface-charged TO-CNF aerogels function well under acidic aqueous and organic media, protonation has been proven to be highly effective in reducing inter-CNF electrostatic repulsion to facilitate compact, tenaciously wet-stable, and pH-stable aerogels.

### Compressive Behaviors

**Figure 6:**
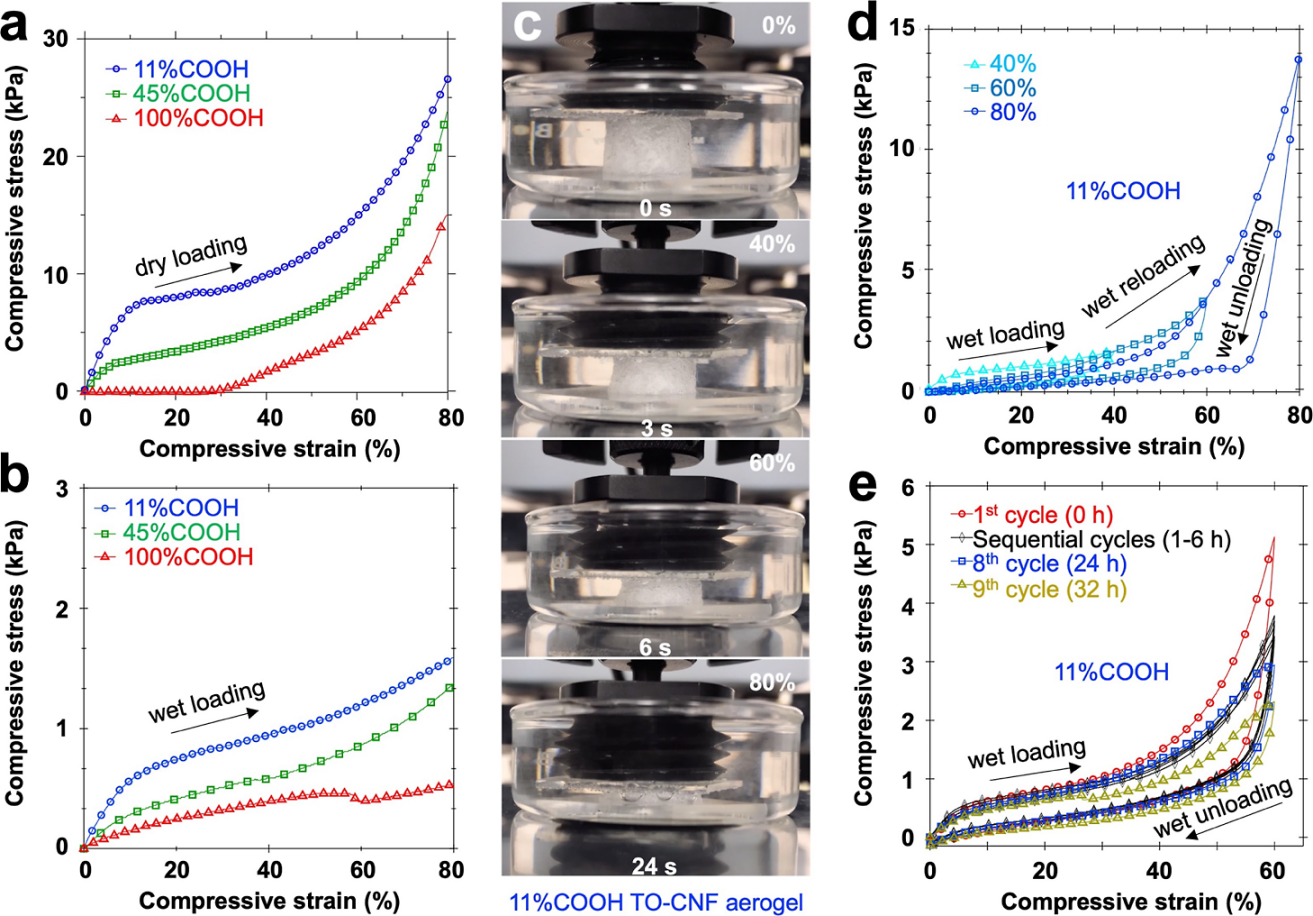
Uniaxial stress–strain compression of protonated TO-CNF aerogels: (a) dry loading; (b) wet loading; (c) incremental wet loading–unloading of the 11% COOH TO-CNF aerogel; and (d) cyclic wet loading–unloading of the 11% COOH TO-CNF aerogel.

The densest (8.0 mg/cm^3^) aerogel from the most charged 11% COOH TO-CNF showed a dry compression modulus of 11.7 kPa/(mg/cm^3^) (ε, 1–2%) (Table 2) and a stress–strain loading curve with three regimes, i.e., initial linear elastic behavior (ε < 10%), plastic deformation (ε, 10–30%), and densification (up to ε = 80%) (Figure 6a). The less-dense aerogels from partially and fully protonated TO-CNFs had significantly lower moduli of 8.2 and 1.5 kPa/(mg/cm^3^), respectively, accompanying deformation over wider strain regions. Notably, it was only upon densification (ε > 30%) that the least dense 100% COOH TO-CNF aerogel showed stress. The relative moduli of these aerogels were proportional to their relative density, or , where their respective scaling factor (*n*) was 2.73, 2.84, and 2.92 or an inverse density (ρ) and modulus (*E*) relationship typical of honeycomb-like cellular structures.^32^ The compression strength at maximum loading (ε = 80%) also lowered with charge-dependent density reduction from 3.6 kPa/(mg/cm^3^) to 2.9 kPa/(mg/cm^3^) for the respective as-is and fully protonated aerogels, although it was far less affected than the moduli. The effect of protonation on compressive properties was measured by the effect size,^33^ which indicates the relationship between the mediating variable, density, and the outcome, moduli or strength, of the aerogels (Table 2). The larger the effect size, the greater the magnitude of the difference between the average dry moduli and strengths, i.e., while both lowered significantly with decreasing density, the dry moduli experienced the most significant reduction (*f* = 7.07, *r* = 0.998, *p* < 0.001).

**Table 2:** Dry and Wet Compression Properties of Aerogels from TO-CNFs with Three Levels of Protonation: Assessment of the Influence of Density on Modulus and Strength by ANOVA

independent variable	mediating variable	outcomes								
COOH	density	dry Young’s modulus			dry strength		wet Young’s modulus		wet strength	
		(ε = 1–2%)			(ε = 80%)		(ε = 1–2%)		(ε = 80%)	
(%)	(mg/cm^3^)	(kPa)	[kPa/(mg/cm^3^)]	*n*	(kPa)	[kPa/(mg/cm^3^)]	(kPa)	[kPa/(mg/cm^3^)]	(kPa)	[kPa/(mg/cm^3^)]
11	8.0	93.3	11.7	2.73	28.5	3.6	26.5	3.3	12.9	1.7
	(±0.6)	(±3.1)			(±2.1)		(±2.9)		(±4.6)	(±0.6)
45	6.6	53.9	8.2	2.84	24.6	3.7	14.3	2.2	11.9	1.8
	(±0.8)	(±8.1)			(±0.6)		(±0.0)		(±2.2)	(±0.3)
100	5.2	7.7	1.5	2.92	15.1	2.9	7.1	1.4	5.1	1.1
	(±0.8)	(±0.0)			(±4.9)		(±0.0)		(±1.6)	(±0.2)
effect size (*f*)^a^	1.62	7.02			2.52		5.32		0.66	
		(large)			(large)		(large)		(large)	
*p* value^b^	0.000	0.000			0.050		0.001		0.018	
*r*	0.991	0.998			0.972		0.989		0.919	

a The effect sizes with an *f*-value >0.4 were considered significant effects.^33^

b Coefficients with a *p*-value <0.05 were statistically significant by one-way ANOVA.

All three aerogels showed significantly reduced wet compression modulus and strength (Table 2, Figure 6b), which was expected due to the plasticizing effect of absorbed water in the inter-nanofibril regions,^8^ and is consistent with Eshelby’s theory of a dry foam softened upon wetting by the inclusion of a high surface tension liquid^34,35^ and mediated by protonation-dependent density. The least indiscernible dry and wet moduli of the 100% COOH TO-CNF aerogel confirmed the negligible plasticization effect of water. Furthermore, they confirmed that protonation eliminated electrostatic inter-TO-CNF repulsion, enabling the most extensive self-assembly of TO-CNFs in the cellular walls. Hence, protonation-dependent density changes were ascribed to reduced electrostatic repulsion between glass and surface-charged CNFs. In the absence of charges, ice crystals grew larger and the excluded volume diminished, giving aerogels with larger pores and CNFs that associated more tenaciously into thinner and more compact cellular walls.

Conversely, the decane-saturated as-is 11% COOH TO-CNF aerogel was a hardened gel as in a compliant matrix stiffened by liquid inclusion.^34^ Since liquid absorption was primarily driven by surface wetting and capillary flow in the pore structure, the rigid organo-gel was attributable to the more hydrophilic cellular surfaces of the 11% COOH TO-CNF aerogel, which absorbs more water than decane or chloroform (Table 1). Hence, the stiffening of the as-is aerogel in decane was ascribed to a repulsive hydrophobic effect, while the softening in water was a plasticizing effect. As a result, evaporative air drying of both decane- and chloroform-saturated aerogels returned both with full shape retention, while strong meniscal forces collapsed the water-saturated aerogel into a film-like structure.

Incremental cyclic compressive loading in water (pH 5.7) of the as-is aerogel released air bubbles underneath the upper glass plate (Figure 6c), evident in the opening of some closed cells (Figure 5a,b). Stepwise loading–unloading in the water from 0% to 40, 60, and 80% compressive strain showed distinct linear elastic regions and full shape recovery hysteresis upon unloading (Figure 6d). Cyclic wet compressions of the as-is aerogel from 0 to 60% strain, i.e., the first seven cycles hourly and the eighth cycle at 24 h and the ninth cycle at 32 h showed decreasing strength from an initial 5.1 kPa (1^st^ cycle) to 2.9 kPa by the eighth cycle and then lastly to 2.3 kPa (Figure 6e). The aberration at ε ∼ 25% loading during the ninth cycle after 32 h indicated structural buckling of the cellular structure, which was consistent with the loss of structural integrity after 48 h of static immersion at pH 5.7 (Figure 5e).

Furthermore, the as-is TO-CNF aerogel was weaker than a C2-C3 carboxylated CNF aerogel from sequential periodate and chlorite oxidation of the same RS cellulose, i.e., a 50.2 kPa(mg/cm^3^) dry compressive modulus and 8.2 kPa(mg/cm^3^) strength,^3^ but similar to a hybrid 90/10 carboxymethyl CNF/alginate aerogel [12 kPa(mg/cm^3^)]^36^ yet more robust than a cross-linked carboxymethyl CNF aerogel [8.4 kPa(mg/cm^3^)].^29^ As for wet strength, the as-is aerogel with a 3.3 kPa(mg/cm^3^) wet modulus was higher than the C2-C3 RS aerogel, ascribed to the more symmetric lateral dimension and stereoregularity of C6 carboxylate surfaces than C2-C3 dialdehyde/dicarboxylate surfaces, while weaker than the cross-linked uncharged CNF aerogel [5.7 kPa(mg/cm^3^)].^19^ Even the fully protonated aerogel had a higher wet modulus [1.4 kPa(mg/cm^3^)] than the cross-linked microfibril aerogel [0.2 kPa(mg/cm^3^)],^37^ suggesting superior bottom-up self-assembly of CNFs and contributing to our understanding and development of aerogels.^38^

## Conclusions

This work uses the electrostatic repulsion of varying surface-charged TO-CNFs (1.38 nm thick, 2.49 nm wide, ca. 1 μm long; 1.17 mmol/g carboxylation) to elucidate the effect of charge on inter-TO-CNF associations and self-assembly via ice-templating into aerogels. Reducing CNF surface charges by protonation most prominently produced the least dense (5.2 mg/cm^3^), most porous (196 mL/g), and best shape-retained aerogels with the most homogeneous and open pore structure and the smoothest external surfaces. Irrespective of protonation levels, all three aerogels were amphiphilic, super-absorptive, and stable at pH 2 for up to 30 days. The aerogel from uncharged CNF absorbed water, decane, and chloroform equally and in highest quantity, whereas that from the most charged CNF absorbed the least but 20–32% more water than the organics. Despite being the most rigid and strongest aerogels in the air and in water by the scaling relationship, embedding those accessible surface charges from the most charged TO-CNFs into the less packed, and thus thicker cellular walls, resulted in the densest aerogel with the lowest water and pH stability.

Conversely, the absence of electrostatic repulsion from the most protonated and unchanged TO-CNF maximized their proximity to assemble compactly into much thinner and more crystalline cellular walls, confirming the central hypothesis that reducing charged surfaces and interfaces during ice-templating and lyophilization minimizes interfacial spaces and enhances the close and compact associations of TO-CNFs. These findings demonstrate that the porous structure and absorbent and compressive properties of hierarchically self-assembled nanocellulose materials can be tuned and enhanced by aqueous protonation of charged TO-CNFs. The aqueously stable hydrogel and organically stiffened organo-gel also demonstrate the potential for water-based applications and further chemical functionalization or processing to produce new materials for advanced performance end uses.
